# Draft Reference Genome Sequence of *Corynebacterium mastitidis* 16-1433, Isolated from a Mouse

**DOI:** 10.1128/genomeA.00050-18

**Published:** 2018-02-15

**Authors:** Christopher Cheleuitte-Nieves, Christopher A. Gulvik, Ben W. Humrighouse, Melissa E. Bell, Aaron Villarma, Lars F. Westblade, Neil S. Lipman, Vincent A. Fischetti, John R. McQuiston

**Affiliations:** a Center of Comparative Medicine and Pathology, Memorial Sloan Kettering Cancer Center and Weill Cornell Medicine, New York, New York, USA; b Special Bacteriology Reference Laboratory, Bacterial Special Pathogens Branch, Division of High- Consequence Pathogens and Pathology, Centers for Disease Control and Prevention, New York, New York, USA; c Department of Pathology and Laboratory Medicine, Weill Cornell Medicine, New York, New York, USA; d Department of Medicine, Division of Infectious Diseases, Weill Cornell Medicine, New York, New York, USA; e Laboratory of Bacterial Pathogenesis and Immunology, The Rockefeller University, New York, New York, USA

## Abstract

We report here a nearly complete draft genome sequence for a *Corynebacterium mastitidis* isolate from a mouse. The total read coverage is 198×, and the genome size is 2,264,319 bp with a 69.04% GC content. This genome complements the only other genome available for *C. mastitidis*, which was obtained from a sheep.

## GENOME ANNOUNCEMENT

*Corynebacterium* species are small, pleomorphic Gram-positive bacteria with coccoid, club, and rod forms (1). Many members of the genus are commensal and colonize the skin and mucous membranes of mammals. They are known to cause opportunistic infections in mammals, particularly immunocompromised subjects. *Corynebacterium mastitidis* was first isolated from the milk of a sheep (*Ovis aries*) with subclinical mastitis and was subsequently identified as part of the normal microbiota of the ocular surface in humans (2, 3). *C. mastitidis* is reported to play a key role in the development of suppurative inflammation and preputial gland abscesses associated with fight wounds in mice (*Mus musculus*) (1). It has also been identified as an ocular commensal in C57BL/6 mice that drives the release of antimicrobials into the tears to protect the eye from pathogens such as *Candida albicans* and *Pseudomonas aeruginosa* (3). The isolate described here, *C. mastitidis* strain 16-1433, was cultured from a 7-month-old female C57BL/6 mouse that presented with microphthalmia, blepharitis, and keratitis. Aerobic culture of the right eye was performed and grew *C. mastitidis* identified using matrix-assisted laser desorption ionization–time of flight mass spectrometry (MALDI-TOF MS).

The isolate was cultured on tryptic soy agar supplemented with soy lecithin and polysorbate 80 (prepared in-house at the Centers for Disease Control and Prevention) and incubated at 37°C for 48 h. Genomic DNA was extracted according to the Quick-DNA fungal/bacterial micro prep kit (Zymo Research, Irvine, CA, USA) protocol, and the library was created using the NEBNext Ultra DNA kit (New England BioLabs, Ipswich, MA, USA) and quantified using the Qubit version 1.0 fluorometer (ThermoFisher Waltham, MA, USA). Paired-end sequencing (2 × 250 bp) was performed with the Illumina MiSeq platform (Illumina, San Diego, CA, USA). PhiX was removed from the FastQ read files with BBDUK version 37.02 using a 31-mer search allowing for a single nucleotide difference in the query, and Trimmomatic version 0.36 was used to remove adapter sequences and discard low-quality nucleotides (4). Cleaned sister reads along with cleaned broken (singleton) reads were provided to SPAdes version 3.11.1 for *de novo* assembly using the “--only-assembler” option (5). To refine the genome, BWA-MEM version 0.7.16a-r1181 was used to map only the cleaned paired reads back onto the assembly with the “-x intractg” option (6), and SAMtools version 1.3.1 generated a binary alignment map (BAM) file (7). The BAM and assembly files were provided to Pilon version 1.22 and “--fix snps,indels” and “--mindepth 0.5” options were invoked to correct initial assembly errors (8). Two subsequent rounds of polishing were performed with the same parameters to correct errors that were missed due to stringent read mapping parameters and conservative correction. According to QUAST version 4.6.0 and CheckM version 1.0.8, the quality of our genome exceeds the only other assembly (GenBank accession no. NZ_AQXB01000000; 99.97% completeness) available for the species with 100% estimated completeness from 585 single-copy gene markers in the *Corynebacterium* genus (9, 10). The genome of *C. mastitidis* 16-1433 consists of 2,264,319 bp with a GC content of 69.04%, as well as 2,132 protein-coding genes, 51 tRNAs, and 57 RNAs.

### Accession number(s).

This whole-genome shotgun project has been deposited in DDBJ/ENA/GenBank under the accession no. PJAF00000000. The version described in this paper is the first version, PJAF01000000.
